# Mitochondria-Ros Crosstalk in the Control of Cell Death and Aging

**DOI:** 10.1155/2012/329635

**Published:** 2011-11-14

**Authors:** Saverio Marchi, Carlotta Giorgi, Jan M. Suski, Chiara Agnoletto, Angela Bononi, Massimo Bonora, Elena De Marchi, Sonia Missiroli, Simone Patergnani, Federica Poletti, Alessandro Rimessi, Jerzy Duszynski, Mariusz R. Wieckowski, Paolo Pinton

**Affiliations:** ^1^Department of Experimental and Diagnostic Medicine, Section of General Pathology, Interdisciplinary Center for the Study of Inflammation (ICSI), Laboratory for Technologies of Advanced Therapies (LTTA), University of Ferrara, 44121 Ferrara, Italy; ^2^Department of Biochemistry, Nencki Institute of Experimental Biology, 02-093 Warsaw, Poland

## Abstract

Reactive oxygen species (ROS) are highly reactive molecules, mainly generated inside mitochondria that can oxidize DNA, proteins, and lipids. At physiological levels, ROS function as “redox messengers” in intracellular signalling and regulation, whereas excess ROS induce cell death by promoting the intrinsic apoptotic pathway. Recent work has pointed to a further role of ROS in activation of autophagy and their importance in the regulation of aging. This review will focus on mitochondria as producers and targets of ROS and will summarize different proteins that modulate the redox state of the cell. Moreover, the involvement of ROS and mitochondria in different molecular pathways controlling lifespan will be reported, pointing out the role of ROS as a “balance of power,” directing the cell towards life or death.

## 1. Introduction

From the first observation of the real mitochondrial structure in 1950 [1], it was clear that this particular composition reflected the different functions of this organelle. Mitochondria are delimitated by double-membrane architecture, an outer membrane of eukaryotic origin and an inner membrane characterized by the absence of cholesterol and the presence of cardiolipin, typical elements of bacterial membranes. The inner membrane is organized in characteristic folds, termed cristae, which protrude into the matrix and accommodate the respiratory chain complexes. In healthy cells, the inner membrane is impermeable to ions [2], which allows the electrons transport chain (ETC) to actively build up the proton gradient. The mitochondrial membrane potential (ΔΨm) results from the difference in electrical potential generated by the electrochemical gradient across the inner membrane. Through oxidative phosphorylation, mitochondria play their essential role to supply the cell with metabolic energy in the form of ATP. As a consequence, they are also the primary source of cellular reactive oxygen species (ROS), especially at level of the respiratory chain complexes I and III, where electrons derived from NADH and ubiquinone can directly react with oxygen or other electron acceptors and generate free radicals [3, 4]. Therefore, ROS are a normal side product of the respiration process, and they react with lipids, protein, and DNA, generating oxidative damage. Indeed, mitochondria are the major site of ROS production, but also the major targets of their detrimental effects, representing the trigger for several mitochondrial dysfunctions. In this review, we will focus on this deadly liaison, with particular attention on ROS production, mitochondrial ROS targets, and their role in apoptosis, autophagy, and aging.

## 2. Mitochondrial ROS Production

Mitochondria are responsible for 90% of the energy that cells, and thus tissues, organs, and the body as a whole need to function. Hence, they are known as the “cells powerhouse,” the core of cellular energy metabolism, being the site of most ATP generation through mitochondrial oxidative phosphorylation (OXPHOS) [5].

**Figure 1:**
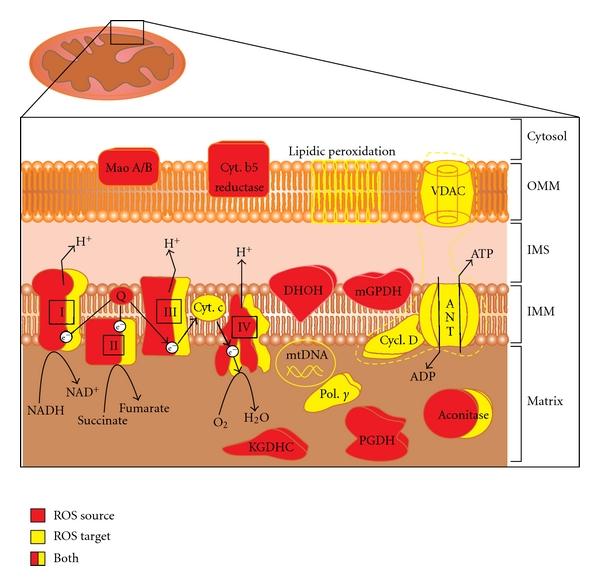
Mitochondrial sources of ROS and mitochondrial ROS targets. ROS generators (red) and ROS targets (yellow) are shown in their precise localizations inside mitochondria. Dotted yellow trace encloses the permeability transition pore components. See text, in particular sections 2 and 3, for further details. Abbreviations: OMM: outer mitochondrial membrane; IMS: intermembrane space; IMM: inner mitochondrial membrane; MAO A/B: monoamine oxidases A and B; Cyt. b5 reduct.: cytochrome b5 reductase; DHOH: dihydroorotate dehydrogenase; mGPDH: glycerol-3-phosphate dehydrogenase; I, II, III, and IV: Complex I to IV of the respiratory chain; Q: coenzyme Q; Cyt. c: cytochrome c; KGDHC: *α*-ketoglutarate dehydrogenase complex; PGDH: pyruvate dehydrogenase complex; e^−^: electrons; VDAC, voltage-dependent anion channel, Cycl. D: cyclophilin D; ANT: adenine nucleotide translocase; Pol. Γ: polymerase *γ*; mtDNA: mitochondrial DNA.

In this process, electrons liberated from reducing substrates are delivered to O_2_ establishing an electrochemical gradient used to drive ATP synthesis. During the OXPHOS, the reduction of oxygen by one electron at a time (O_2_ → O_2_^−^• → H_2_O_2_ → •OH → H_2_O) produces ROS, relatively stable intermediates with one unpaired electron [6–8]. Although Brown and Borutaite in their recent review have presented a number of examples supporting the point of view that mitochondria are not a major source of ROS in the cell [9], the fact that oxidative phosphorylation accounts for 90% to 95% of cellular oxygen consumption and that 3% from that pool can be converted to superoxide is a very strong argument in favour of mitochondria as a main source of this oxygen radical. Hence, it is a firm paradigm that mitochondria are also the major source of ROS in mammalian cells (Figure 1).

The primary ROS generated by mitochondria, as a result of monoelectronic reduction of O_2_, is superoxide anion (O_2_^−^•) that is the precursor of most ROS and a mediator in oxidative chain reactions. In vivo, O_2_^−^• is produced both enzymatically by NADPH oxidase, cytochrome P450-dependent oxygenases, and xanthine oxidase and non-enzymatically, when a single electron is directly transferred to O_2_ [8]. In addition, O_2_^−^• may react with other radicals including nitric oxide (NO•) producing reactive nitrogen species (RNS) [10]. Dismutation of O_2_^−^•, either spontaneously [11], or through a reaction catalyzed by superoxide dismutases (SODs) [12], produces hydrogen peroxide (H_2_O_2_) [13]. H_2_O_2_ generated in mitochondria has many possible fates. Because H_2_O_2_ is relatively stable and membrane permeable, it can diffuse within the cell and be eliminated by cytosolic or mitochondrial antioxidant systems such as catalase, glutathione peroxidase, and thioredoxin peroxidase [14]. Mitochondrially generated H_2_O_2_ can also act as a signaling molecule in the cytosol, affecting multiple networks that control, for example, cell cycle, stress response, energy metabolism, and redox balance [15–17].

When not metabolized, H_2_O_2_ can be further transformed to hydroxyl radical (•OH), in the presence of metal ions, by Fenton reaction [18]. •OH is one of the strongest oxidants in nature, is highly reactive, and generally acts essentially as a damaging molecule. For this reason, mitochondria are believed to have developed efficient H_2_O_2_ removal systems, as well as metal-chelating mechanisms, such as chaperone proteins, preventing the formation of this radical.

At least, ten mammalian mitochondrial enzymes contribute to ROS production [17, 19] even if their capacity of ROS producing greatly differ in a tissues-specific manner [20]. *In vitro* experiments demonstrate that mitochondria isolated from mouse heart, brain, and kidney have selective substrate and inhibitor preferences for H_2_O_2_ generation and that the apparent sites of H_2_O_2_ generation are both substrate and organ specific [21].

The major bulk of mitochondrial ROS generation occurs at the electron transport chain, as a byproduct of respiration [7, 8, 22]. Cytochrome c, oxidase (Complex IV) is the terminal component of the ETC, receives four electrons from cytochrome c and reduces one O_2_ molecule to two H_2_O. It retains all partially reduced intermediates until full reduction is achieved and does not seem to release these intermediates in measurable quantities [6].

Historically, the first mitochondrial site producing ROS was identified at the Complex III located at the inner side of inner mitochondrial membrane (bc1 complex, ubiquinone: cytochrome c reductase). The primary ROS produced at this site is O_2_^−^•, through the referred Q-cycle [23]. Despite the recent advances in understanding the structure of the bc1 complex, a mechanism of O_2_^−^• production is not yet known [24–26]. 

Succinate dehydrogenase (Complex II, SDH) is a flavoprotein located at the inner surface of inner mitochondrial membrane. Although oxidation of succinate can theoretically produce ROS with a high rate, significant O_2_^−^• formation from this enzyme has not been measured so it is still unclear whether SDH produces ROS *in situ*, in mitochondria. Despite the lack of ROS formation by Complex II itself, succinate is an important source of ROS in many tissues through a mechanism involving reverse electron transfer. This particular phenomenon may result from high mitochondrial membrane potential, which thermodynamically favourites electron donor from Complex II to I. Because of this, succinate may promote ROS generation at Complex I level [6, 7].

Mitochondrial Complex I, “NADH dehydrogenase,” in the inner face of inner membrane provides a major entry point for electrons into respiratory chain. It is a significant source of ROS [27] in particular O_2_^−^• [28] and H_2_O_2_ although whether or not Complex I is the major site of ROS production in intact mitochondria *in vivo *is a complicated issue. Indeed all the evidence on ROS production by Complex I was obtained *in vitro *with isolated mitochondria [29]. 

There are other mitochondrial nonrespiratory chain enzymes that produce ROS although pinpointing their specific contribution to total mitochondrial ROS production remains unclear.

Mitochondrial cytochrome b5 reductase is located in the outer mitochondrial membrane [30]. The enzyme is widely distributed in mammalian tissues and may be involved in O_2_^−^• generation with a very high rate ~300 nmoL per min per mg protein [31]. 

Monoamine oxidases (MAO-A and MAO-B) are also located in the outer mitochondrial membrane and ubiquitously expressed in various mammalian tissues. These enzymes catalyze the oxidation of biogenic amines accompanied by the release of H_2_O_2_ [32]. 

Dihydroorotate dehydrogenase (DHOH) is located at the outer surface of inner mitochondrial membrane. It catalyzes the conversion of dihydroorotate to the pyrimidine base, orotate, which is a step in the de novo synthesis of uridine monophosphate. The DHOH is ubiquitously distributed in mammalian tissues, and it is considered a mitochondrial source of O_2_^−^• and H_2_O_2_ although the capacity of DHOH to produce O_2_^−^• requires further clarification [33].

Mitochondrial dehydrogenase of *α*-glycerophosphate (Glycerol-3-Phosphate Dehydrogenase, mGPDH) is located at the outer surface of inner mitochondrial membrane. The mGPDH is involved in lipid metabolism and in the so-called glycerol phosphate shuttle capable of regenerating cytosolic NAD+ from the NADH formed in glycolysis. It is ubiquitously but unequally expressed in various mouse tissues and mediate the production of H_2_O_2_ [34, 35].

Mitochondrial aconitase is an enzyme localized to the matrix space of mitochondria; it participates in tricarboxylic acid cycle catalyzing a conversion of citrate to isocitrate. The enzyme contains an iron-sulfur cluster that can be oxidized by O_2_^−^• or H_2_O_2_ producing •OH [36]. 

Ketoglutarate dehydrogenase complex (KGDHC, 2-oxoglutarate dehydrogenase) is an integral mitochondrial enzyme tightly bound to the inner mitochondrial membrane on the matrix side [37]. It catalyzes the oxidation of *α*-ketoglutarate to succinyl-CoA in the tricarboxylic acid cycle. Structurally, KGDHC is composed of three enzymes: *α*-ketoglutarate dehydrogenase (E1 subunit), dihydrolipoamide succinyltransferase (E2 subunit), and lipoamide dehydrogenase (E3 subunit). The E3 component appears to be the principal ROS source generating O_2_^−^• and H_2_O_2_ [38, 39].

Pyruvate dehydrogenase complex (PGDH) localized into mitochondrial matrix contains flavoenzyme dihydrolipoyl dehydrogenase subunits like KGDHC, which are very important ROS sources [38].

## 3. Mitochondrial Targets of ROS

A wide range of mitochondrial ROS-induced damages has been described, including protein carbonylation, lipid peroxidation, or mtDNA damage. These damages, either individually or collectively can lead to a cellular energetic catastrophe. In this section, we are going to treat the most important mitochondrial targets of ROS action, also summarized, with their mitochondrial localization, in Figure 1.

### 3.1. Oxidative Damage of mtDNA

The sequence and organization of the human mitochondrial genome has been presented for the first time by Anderson et al. in the landmark paper in 1981 [40]. Mitochondrial DNA (mtDNA) was identified as a 16.5 kb circular molecule encoding 13 polypeptides, 22 tRNAs, and 2 rRNAs. All of the above mentioned are components of mitochondrial respiratory machinery. MtDNA is attached to the IMM on the matrix side, rendering it particularly prone to oxidative damage, as the aforesaid localization is in immediate proximity to the cells' major source of ROS, that is to the mitochondrial respiratory chain. ROS accumulating within the matrix are in such a case explicitly deleterious. Additionally, the lack of introns, histones, and other DNA-protecting proteins as well as DNA repair systems inferior to those observed in nDNA allows increased deterioration [41, 42]. However, mitochondria possess a number of DNA repair enzymes such as MYH (2-OH-A/adenine DNA glycosylase) and OGG1 (8-oxoG DNA glycosylase) [43]. In nucleic acids, both the bases as well as the sugar are prone to ROS attacks. This may lead to modifications of base and abasic sites and result in strand breaks [44]. A good example that may be the addition of the hydroxyl radical to the double bonds may occur. In this case the reaction rate-limiting factor is diffusion of approximately 4.5 × 10^9^ to 9 × 10^9^ M^−1 ^s^−1^ [45]. The main outcome of the above-mentioned attacks is the hydroxylation of pyrimidines at C5 and C6 as well as purines at C4, C5, and C8. So far over 80 different oxidized bases in DNA exposed to oxidant factors, such as H_2_O_2_ and singlet oxygen, have been identified [45, 46]. Experiments from the mid 1980s led to the establishment of a dependable marker of these alterations—8-hydroxy-deoxyguanosine (8-OHDG) [47–51]. An increase in 8-OHDG is a sign of mtDNA fragmentation and is observed in events involving oxidative stress [52].

Alterations in the mitochondrial genome are reflected in a number of disorders, most of which involve impaired respiration and oxidative phosphorylation. As a matter of fact, over 150 pathogenic mutations have been identified in the mtDNA, which cause human diseases [53]. The scope of these disorders has been positively correlated with increasing age and tissues with high-energy demands [54–58]. Individuals may develop a number of symptoms including ataxia, dystonia, epilepsy, dementia, peripheral neuropathy, optic atrophy, and deafness [59]. A number of authors report associations between mtDNA point mutations and neurodegenerative diseases [60–63]. However, some of the mutations are yet to be confirmed and therefore are under dispute. On the other hand, in some cases, such as Parkinson's disease (PD), there seems to be a consistent PD-associated haplogroup [64–66]. Brains of aged individuals as well as those with neurodegenerative diseases exhibit increased levels of mutated mtDNA, particularly in substantia nigra neurons, as compared to hippocampal neurons [67]. It is not clear, whether these mutations are an effect of oxidative stress. Nevertheless, such explanation seems reasonable.

### 3.2. Oxidative Damage of Mitochondrial Proteins

Oxidative modification of proteins (oxidation of arginine, lysine, threonine, and proline residues) induces the formation of protein carbonyls, commonly detected under oxidative conditions, in aged cells and in aged animal tissues. On the other hand, peroxynitrite oxidizes tryptophan, cysteine, and tyrosine protein residues as well as unsaturated fatty acids and low molecular mass components of the antioxidant defense system like glutathione, *α*-tocopherol, and ascorbate. Additionally, oxidative damage can affect carbohydrates, which, when oxidized, may be used in glycation process causing the formation of glycation end products. It has been repeatedly shown that the accumulation rate of such oxidatively modified proteins (under oxidative stress or in aging) is tissue specific and depends on protein type [68].

#### 3.2.1. Mitochondrial Respiratory Chain

The mitochondrial respiratory chain, due to its natural imperfections, can be considered a peculiar superoxide generator. In such a case, complexes of the respiratory chain are in the firsthand exposition to oxidative modification because they are present at the sites of ROS production. The high sensitivity of the respiratory chain complexes to oxidizing agents results from the fact that they contain iron-sulfur clusters (Complexes I, II, and III), heme groups (Complexes II, III, and IV) and copper centers in complex IV, all of which can be a site of direct ROS attack, and their oxidative modifications can manifest with a decrease of their enzymatic activity and dysfunction of the whole respiratory chain [69–71]. Interestingly, three mechanisms have been reported in the NO• inhibition of Complex I: S-nitrosation, tyrosine nitration and damage to FeS centres [72]. The inhibition of mitochondrial Complex I activity is consistently detected in Parkinson's disease patients as well as in mitochondrial toxin models of the disorder [73].

Cytochrome c (a component of OXPHOS) can be modified by nitration of its tyrosine residues, which also affects electron flow through the respiratory chain [74]. Nitration of a single tyrosine residue in cytochrome c by a relatively low dose of peroxynitrite resulted in the upregulation of its peroxidase activity for H_2_O_2_ and in the impairment of the membrane potential formation [75]. Oxidative damage of individual respiratory chain complexes results not only in a decreased efficiency of ATP production but can also increase ROS production by oxidatively affected OXPHOS subunits, contributing in this way to the intensification of oxidative stress.

#### 3.2.2. Mitochondrial Carriers

The effect of oxidative stress (i.e., caused by site-specific metal-catalyzed oxidation) on mitochondrial carriers was studied intensively in a model exploiting components of the mitochondrial permeability pore: the adenine nucleotide translocase (ANT), porin (VDAC), and creatine kinase. ANT contains many proline, arginine, and lysine residues, which are very sensible for such type oxidation (systems: vanadyl/H_2_O_2_-generating hydroxyl free radicals or Cu^2+^/tert-butyl hydroperoxide). Moreover, ANT is localized in the IMM in cardiolipin-rich (containing unsaturated fatty acids) domains. This can explain its sensibility to oxidation also by lipid peroxidation products. In 1998, interesting studies of Sohal's group revealed that ANT in mitochondria of flight muscles is significantly carbonylated in senescent flies. Surprisingly, ANT was the only mitochondrial protein demonstrating age-related increase of carbonylation in this experimental model. In vitro experiments basing on the exposure of isolated mitochondria to the hydroxyl free radicals showed an increased level of carbonylated ANT exhibiting lower nucleotide exchange activity [76].

In addition to controlling the release of superoxide from mitochondria [77], VDAC, which accounts for about 10% of outer mitochondrial membrane (OMM) proteins, can also be a target for intracellular O_2_^−^•. Madesh and Hajnoczky observed that VDAC-dependent permeabilization of the OMM and cytochrome c release can be caused by superoxide but not by H_2_O_2_. Moreover, the observed effect of superoxide does not require the contribution of the proapoptotic proteins like Bak or Bax [78]. On the other hand, another component of the mitochondrial permeability transition pore (mPTP), mitochondrial creatine kinase, present in the intermembrane space (IMS) is affected by H_2_O_2_ and peroxynitrite. The drop in the creatine kinase activity is caused by oxidative modifications of its sulfhydryl groups and aromatic aminoacids [79, 80]. 

#### 3.2.3. Permeability Transition Pore as a Ros Target

Based on the literature, permeability transition pore (mPTP) seems to be a multiprotein complex with variable composition. The main core of mPTP consists of the voltage-dependent anion channel (VDAC), ANT, and cyclophilin D localized in the outer-, inner mitochondrial membrane, and mitochondrial matrix, respectively [81–83]. Still there is no consensus concerning exact composition of mPTP. Recent studies with the use of transgenic VDAC1 or ANT1 knockout mice seem to contradict the stereotype model of PTP. To find more about the presence of VDAC, ANT, or cyclophilin D in the mPTP, refer to [84–86]. The other components, such as hexokinase, benzodiazepine receptor, and creatine kinase seem to play a regulatory role rather than being structural components of the mPTP. However, regardless of the composition of mPTP, it has been repeatedly described already in 90ties that oxidation of critical thiol residues of ANT induces opening of the mPTP and collapse of mitochondrial membrane potential, mitochondrial swelling, and cytochrome c release. When the thiol groups of ANT are in reduced state, probability of mPTP opening is much lower than when they are oxidised and cross-linkage of ANT thiol groups can occur. Especially oxidation of the Cys^160^ and Cys^257^ of ANT promotes PTP opening [87]. Moreover, Cys^56^ oxidation alters the conformation of ANT inducing it to become a nonspecific pore, thus mimicking the effect of Ca^2+^ which converts ANT into a large channel [88]. Sensitivity of ANT to the oxidizing agents makes mPTP one of the direct sites of ROS action in mitochondria. 

#### 3.2.4. Aconitase

In Section 2, we have argued m-aconitase as a source of ROS, but, at the same time, it can be considered a well-established example of oxidative damage targeting a mitochondrial enzyme. In fact, its susceptibility to oxidative damage mainly from superoxide is related to the iron-sulfur cluster present in the active site of the enzyme [89].

#### 3.2.5. Polymerase *γ*

Nearly 10 years ago, the studies of Copeland's group confirmed that polymerase *γ* present in the mitochondrial matrix responsible for mtDNA replication can also be considered as an intrinsic target for ROS. The group found that the catalytic subunit of polymerase *γ* is one of the proteins most abundantly oxidized in the mitochondrial matrix and correlated oxidative modification of polymerase *γ* with the drop of its enzymatic activity. This in turn can result in the reduction of mtDNA repair and replication [90].

### 3.3. Peroxidation of Mitochondrial Lipids

In mitochondrial membranes, unsaturated fatty acids, being components of phospholipids, are very susceptible to oxidation by the hydroxyl radical. Lipid peroxidation products, lipid hydroperoxides, generate very reactive unsaturated aldehydes like 4-hydroxy 2-nonenal (4-HNE), malondialdehyde (MDA), and acrolein. Moreover, the generation of lipid radicals can induce a chain reaction leading to the generation of new radicals which intensifies lipid peroxidation. Lipid peroxides cause various effects in the cell. It has been reported that oxidatively modified lipids affect membrane fluidity; this is the case of lipid peroxidation at inner mitochondrial membrane level, leading to increased permeability to protons and uncoupling of oxidative phosphorylation [91]. Moreover, lipid radicals diffuse easily in the membranes and can covalently modify membrane proteins [92], as well as cause “lipoxidative” damage to the mtDNA. For example, 4-HNE inhibits ANT activity in isolated mitochondria due to the modification of critical sulfhydryl groups in ANT [93]. It has also been suggested that the protein carbonylation process is more often related to the lipid-protein oxidation than the direct protein oxidation by reactive oxygen species [94, 95].

## 4. Mitochondrial ROS Measurements

Direct imaging of ROS in biological samples has proven to be extremely challenging. A large amount of methods for ROS monitoring has been developed in the last years, including fluorimetric, spectrophotometric, and chemiluminescent assays [96].

Among different fluorescent assays, MitoSOX is largely used to visualize superoxide ions inside mitochondria [97]. This reagent is a cell permeant dye that once in mitochondria is oxidized by superoxide and trapped inside organelle, becoming weakly fluorescent. Subsequent binding with nucleic acid permit to the dye to show high fluorescence, with excitation at 510 nm and emission at 580 nm. It can be considered a superoxide-specific probe, because its reactivity for hydrogen peroxide or reactive nitrogen species is relatively low. However, several caveats exist in using MitoSOX probe [98, 99]; one of the mostly frequent is, without doubts, the photochemical oxidation of the dye, an aspect which has to be taken into consideration for proper interpretation of results.

One of the widespread methods to detect H_2_O_2_ production by mitochondria is based on 2′,7′ dichlorofluorescein (DCF-H_2_) oxidation. This dye is commercialized as a diacetate chemical probe, in order to be cell permeant, and, once deacetylated from cellular diesterases, it becomes impermeable and weakly fluorescent. DCF-H_2_, when oxidized by H_2_O_2_, is converted into 2′,7′-dichlorofluorescein (DCF), a high fluorescent component (*λ*_ex_ 500 nm − *λ*_em_ 520 nm) [100]. DCF formation is directly proportional to H_2_O_2_ production; therefore, for data analysis is preferable to calculate variation in fluorophore accumulation speed. One of the most relevant limitations of DCF is that it cannot be used to measure H_2_O_2_ production exclusively inside mitochondria. Mitochondria peroxy yellow 1 (MitoPY1), a new type of fluorophore for imaging mitochondrial H_2_O_2_ in living cells with ROS and spatial specificity [101], may represent the answer to this problem, but further studies have to be performed to clarify its real efficacy.

Recently, a new highly specific fluorescent probe has been introduced in methods for ROS measurements. HyPer (distribuited by Evrogen) is a genetically encoded fluorescent sensor specific for H_2_O_2_ and consists in a circularly permuted yellow fluorescent protein (cpYFP), inserted into the regulatory domain of OxyR, a hydrogen peroxide-sensitive transcription factor isolated from *E. coli*. HyPer is a ratiometric indicator; the excitation wavelength in normal condition is 420 nm, and, in the presence of H_2_O_2_, a red-shift moving excitation to 500 nm occurs. Emission spectrum has a maximum at 516 nm, independently from oxidants activity. The most palatable feature is the possibility to direct HyPer to mitochondrial matrix using COX-targeting sequence, permitting directing fast measurements of mitochondrial ROS production [102]. HyPer is not the first ratiometric probe used for ROS assessment. In 2004, Tsien and coworkers developed ratiometric redox-sensitive versions of GFP, termed redox-sensitive green fluorescent proteins (roGFPs) [103, 104]. When a population of roGFP is oxidized, excitation increases at the 400 nm peak while diminishing at the 480 nm peak; the emission is measured at 510 nm. Because the indicator is genetically encoded, it can be targeted to specific proteins or organelles of interest and expressed in a wide variety of cells and organisms (for an exhaustive discussion of the biophysical properties and development of these probes, see [105]). 

Fluorescent dyes show the high advantage to be easy to use, fast, and weakly invasive, leading to a quite accurate calibration using peroxide (or nitric oxide) donors. However, they are fast degradable and easily produce artefacts; in fact, as cited above, high light intensity could induce the activation of the dye, with consequent artefactual ROS generation, resulting in nonspecific signal amplification. 

## 5. Deadly Liaisons: ROS and Mitochondria in the Control of Cell Death

As mentioned in the introduction, mitochondria are often targets of high ROS exposure with deleterious consequences, such as oxidative damage to mitochondrial DNA. This is certainly true, but recent evidence has suggested a deep involvement of ROS also in the extrinsic pathway of apoptosis.

The extrinsic receptor-mediated death pathway requires effective engagement between the death receptors found on the surface of the cell membranes and their respective ligands [106]. The receptor-mediated pathway involves death receptors from the tumor necrosis factor (TNF) superfamily such as TNF, CD95 (Fas), and TNF-related apoptosis-inducing ligand (TRAIL) receptors. Fas and TNFR1 activation is known to generate ROS in response to stimulation, which has been hypothesized to be due to the production of superoxide (O_2_^•−^) because of the formation of lipid raft-derived NADPH oxidase platforms. This lipid raft-associated ROS downstream generation may be of high importance in induction of apoptosis or necrosis [107, 108]. Moreover, ROS are required for apoptosis induction by Fas ligand (FasL) in primary lung epithelial cells. ROS mediate the downregulation of FLIP (FLICE inhibitory protein, a strong inhibitor of apoptosis) by ubiquitination and subsequent degradation by proteasome or through nitric oxide (NO) scavenging that prevents FLIP S-nitrosation and cytoprotection [109].

Other evidences underline how ROS generation may influence the intracellular milieu, favouring the effective execution of the downstream events leading to extrinsic apoptosis. ROS has been shown to sensitize cancer cells to TRAIL-induced apoptosis [110], and massive upregulation of CD95 and TRAIL death receptors have also been observed in response to hydrogen peroxide, through a mechanism involving the activation of NF-kappaB [111]. Interestingly, the ability of H_2_O_2_ to promote apoptosis, through the activation of JNK, seems inhibited in lung fibroblasts from TNFR1-deficient mice [112]. ROS activation of JNK can induce extrinsic or intrinsic apoptotic signaling. TNF*α* is a potent activator of the MAPK cascade, and TNF*α*-induced ROS cause oxidation and inhibition of JNK-inactivating phosphatases, by converting their catalytic cysteine to sulfenic acid, with a consequent prolonged JNK activation, which is required for cytochrome c release and caspase 3 cleavage, as well as necrotic cell death [113].

Upstream of JNK is the redox-sensitive MAPK kinase kinase, ASK1. Under non-oxidizing conditions, reduced thioredoxin1 (Trx1) binds ASK1; the resultant Trx1/ASK1 complex, called “ASK1 signalosome,” functions as a perfect redox switch. Robust and sustained cellular ROS induce the dissociation of oxidized Trx1 from the complex and let to complete the activation of ASK1, also through the recruitment of TRAF2/6 [114]. Moreover, ASK2, another member of the ASK family, binds and stabilizes ASK1 not only in the cytosol, but also in nucleus and mitochondria. Recently, Saxena et al. have shown how the thioredoxin-interacting protein TXNIP, a ubiquitously expressed redox protein that promotes apoptosis, could shuttle from nucleus to mitochondria under oxidative stress, forming a complex with mitochondrial Trx2 and removing it from ASK1. This alleviation of Trx2-mediated inhibition results in phosphorylation of ASK1 and consequent induction of the mitochondrial pathway of apoptosis, with cytochrome c release and caspase-3 cleavage [115].

The major ROS target inside mitochondria is undoubtedly the permeability transition pore (see Section 3). Oxidative modifications of mPTP proteins will significantly impact mitochondrial anion fluxes [116]. In response to proapoptotic stimuli, including ROS and Ca^2+^ overload, the mPTP assumes a high-conductance state that allows the deregulated entry of small solutes into the mitochondrial matrix along their electrochemical gradient. This phenomenon, which is known as mitochondrial permeability transition (MPT), results in immediate dissipation of the mitochondrial membrane potential and osmotic swelling of the mitochondrial matrix [2]. Mitochondrial swelling is defined as an “increase in the volume of mitochondria due to an influx of fluid.” The mechanism concerns an early phase of mitochondrial swelling, which involves movement of water from the intercristal spaces into the matrix; when this water movement continues, the pressure applied to the outer membrane from the increased matrix volume leads to the opening of mPTP and/or rupture of the mitochondrial outer membrane, allowing further matrix expansion [117]. This leads to the release of cytochrome c and the subsequent engagement of the Apaf-1-pro-caspase 9 apoptosome complex, which activates downstream effector caspases. Cytochrome c is present as loosely and tightly bound pools attached to the inner membrane by its association with cardiolipin; this interaction must first be disrupted to generate a soluble pool of this protein. In 2002, the group of Orrenius elegantly showed how oxidative modification of mitochondrial lipids, specifically cardiolipin, is fundamental for tightly bound pool of cytochrome c mobilization, that is so detached because of disturbances in membrane structure [118]. The cardiolipin-bound cytochrome c assumes the role of a membrane-bound peroxidase that can effectively catalyze oxidative stress and cause oxidation of cardiolipin if a source of oxidizing equivalents, such as H_2_O_2_, is activated. This can lead to a putative “oxidative wave propagation:” membrane-bound cytochrome c may be seen as a mitochondrial death receptor transducing proapoptotic signals into executing oxidative cascades, with a consequent overload of oxidized cardiolipin species, detachment of cytochrome c from the membrane and formation of mPTP (reviewed in [119]). In this regard, it has been recently shown that overexpression of human telomerase reverse transcriptase (hTERT), the catalytic subunit of the telomerase holoenzyme, alleviates cellular ROS levels also through its mitochondrial localization, improving activity of cytochrome c oxidase, blocking cytochrome c release, mitochondrial membrane permeabilization, and inhibiting apoptosis in cancer cells [120].

The pathophysiological importance of intracellular redox balance and apoptosis regulation was also taken in to consideration during the study of cerebral cavernous malformations (CCMs) [121]. Ablation of Krit1, a protein whose loss of function has been associated to this particular pathogenesis, leads to a significant increase in intracellular ROS levels, due to the modulation of the expression of the antioxidant protein SOD2 and to a drastic decrease of mitochondrial energy metabolism, with consequent increased susceptibility to oxidative damage [121]. In the same way, MUC1, an oncoprotein aberrantly expressed in acute myeloid leukemia (AML) cells, regulates ROS levels and the differentiation of hematopoietic cells [122]. MUC1 expression is associated with attenuation of endogenous and H_2_O_2_-induced intracellular levels of reactive oxygen species [123], and, inhibition of MUC1 results in the disruption of redox balance and thereby AML cell death [122].

Redox regulation of proteins by moderate levels of ROS is observed in various signaling pathways, including autophagy, a catabolic pathway for degradation of intracellular proteins and organelles via the lysosome [124]. Autophagy is activated mainly by nutrient starvation, and it plays a dual role; it is primarily a surviving mechanism, but it also leads to cell death (called type III cell death) thus possibly acting as an alternative to apoptosis. It is generally accepted that ROS induce autophagy [125, 126], and that autophagy, in turn, serves to reduce oxidative damage [124]. Given that, antioxidants may represent natural inhibitors of this process. As a matter of fact, the p53-target gene TIGAR contributes to the regulation of intracellular ROS levels by modulation of the glycolytic pathway, increasing NADPH production (with a consequent decrease in intracellular ROS levels) and lowering the sensitivity of cells to p53-dependent apoptosis induced by oxidative stress [127]. In this manner, TIGAR inhibits autophagy induced by nutrient starvation and metabolic stress, in a p53-indipendent way [128]. Moreover, a mutant form of SOD1 (SOD1^G93A^, associated with one-fifth of familial amyotrophic lateral sclerosis cases [129]) promotes ROS accumulation and autophagy [130, 131], despite its role in muscular atrophy induction has not been fully understood. In transgenic mice spinal cord, SOD1^G93A^ interacts with p62, an LC3-binding partner known to target protein aggregates for autophagic degradation, suggesting that p62 may direct mutant SOD1 aggregates to autophagy [132].

Because mitochondria are, at the same time, primary source and target of ROS, they play a fundamental role in ROS-mediated autophagy regulation. Indeed, cells use a specialized form of autophagy, called mitophagy, to selectively eliminate defective mitochondria. The term, coined by Lemasters in 2007 [133], indicates a selective degradating process to maintain a healthy population of mitochondria. Increases in cellular ROS lead to loss of mitochondrial membrane potential (ΔΨm), which is considered a trigger for mitophagy [133]. Under serum deprivation, a typical decrease in mitochondrial membrane potential is observed in hepatocytes prior to engulfment by autophagosomes, whereas Cyclosporin A (CsA), a mPTP inhibitor, prevented this depolarization and the autophagosomal proliferation [134, 135]. However, in different setups represented by rat pituitary GH3 cells [136] and muscles [137], Cyclosporin A works as a strong autophagy-inducer: in particular, CsA treatment in skeletal muscles of collagen VI knockout mice (Col6a1 −/−), characterized by impaired autophagy, presence of abnormal mitochondria, and myofiber degeneration, promotes autophagy with a concomitant block of apoptotic degeneration and recovery of muscle strength [137]. This discrepancy suggests that mitochondrial depolarization is not general, leading event to rage the mitophagic process.

Abnormalities in mitochondrial respiration and increased oxidative stress are observed in cells and tissues from parkinsonian patients [138], which also exhibit increased mitochondrial autophagy [139]. Two genes, which encode the OMM kinase PINK1 and the E3 ubiquitin ligase parkin, are mutated in autosomal recessive Parkinson's disease [140]. Parkin is able to induce mitophagy through translocation from cytosol to mitochondria in many different cellular settings, after stress induction by mitochondrial uncouplers, which mimic damage by decreasing the mitochondrial membrane potential [141], or by the administration of oxidative stress inducers, such as Paraquat [142]. Moreover, Parkin may signal the selective removal of defective mitochondria within the cell. Overexpression of Parkin can eliminate mitochondria with deleterious COXI mutations in heteroplasmic cybrid cells, thereby enriching cells for wildtype mtDNA and restoring cytochrome c oxidase activity [143]. PINK1 (PTEN-induced kinase 1) mediates recruitment of parkin to the damaged mitochondria and activates parkin's ligase activity [144], thereby promoting autophagy. Recently, it has been shown that knockdown of PINK1 expression also leads to mitophagy in SH-SY 5Y cells, due to mitochondrial fragmentation and mitochondrial ROS production [145]. PINK1 knockdown led to decreased transmembrane potential, which was ameliorated by antioxidant MnTBAP treatment, suggesting that ROS production is upstream of mitochondrial depolarization [146].

**Figure 2:**
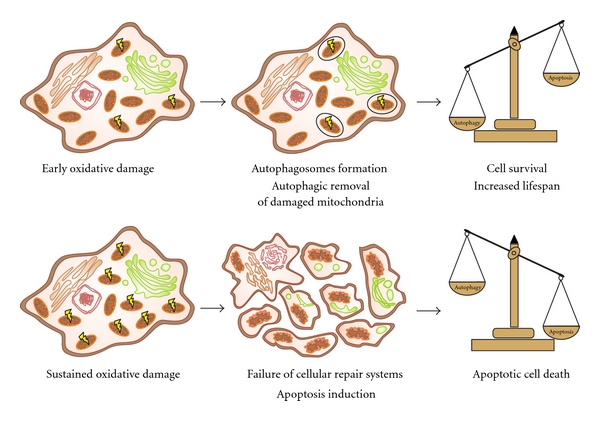
ROS levels control cell fate. Low production of ROS works as trigger of autophagic/mitophagic process, with consequent removal of damaged mitochondria and in turn cellular survival (upper panel). On the other hand, high levels of ROS lead to cell death promoting the apoptotic pathway when prosurvival attempt fails (lower panel).

Based on these observations, ROS act as signaling molecules influencing cell fate. Undoubtedly, redox regulation can promote both survival, during starvation for example, as well as cell death, during oxidative stress. ROS play the role of trigger in the early phase of autophagic process, inducing cytoprotection by eliminating potential sources of proapoptotic stimuli. On the other hand, if the prosurvival attempt fails, ROS cause cell death which involves either the autophagic or the apoptotic pathway, or both (Figure 2).

## 6. ROS as Key Regulators of Aging

The decline associated with aging is caused by the accumulation of ROS, as supported by cellular and biological data from different model systems and organisms [147]. Indeed defects in antioxidant defense mechanisms fail to protect against oxidative damage, reducing lifespan [148, 149] and causing cardiomyophaty, neurodegeneration [150], and cancer [151].

The relationship between mitochondria dysfunctions observed during aging and ROS production is still debated. However, it is clear that the decline of the integrity of mitochondria as a function of age is implicated in aging and age-related diseases [147].

Given this wide scientific evidence, many studies were aimed to identify the molecular mechanisms responsible for ROS deleterious effects on the aging process. Genes that extend lifespan partially included those involved in oxidative stress response, while partially were clustered in the IGF-1/insulin-like signaling pathway [152, 153]. 

A key mitochondrial effector is the adapter protein p66shc, which directly mediate, the production of ROS within the mitochondria [154], participating in intracellular pathways that control oxidative stress, apoptosis, and lifespan determination [155, 156]. Accordingly, p66shc knockout mice are one of the best characterized genetic model of longevity, is being more resistant to oxidative stress [157] and protected against age-dependent, ROS-mediated cardiovascular complications induced by diabetes [158–160]. On the contrary, overexpression of p66shc causes alterations of mitochondrial Ca^2+^ responses (which is an early event of mitochondrial damage) and fragmentation of mitochondrial network [161], leading to cytochrome c release and apoptosis [162]. Orsini and coworkers showed, for the first time, that oxidative stress promotes a translocation of part of the cytosolic pool of p66Shc to mitochondria, binding mtHsp70 in mitochondrial matrix [163]. The same group in 2005 claimed that p66Shc is rather present in the intermembrane space, where it interacts with cytochrome c. This interaction, which results in H_2_O_2_ production [162], can be supported by an earlier report showing that p66Shc has internally located mitochondrial targeting sequence [164]. In order to explain its activity, p66Shc has to be phosphorylated at serine 36 by PKC*β*, and this event leads to a consequent recognition by the prolyl isomerase Pin1, allowing p66Shc entrance into mitochondria [156]. Our recent data have indicated that p66Shc is also present in MAM (mitochondria-associate membranes) fraction, which consists in membranes interacting with mitochondria [165, 166]. Interestingly, the level of p66Shc in MAM fraction changes in an age-dependent manner (MAM fraction isolated from livers of old animals contained more p66Shc than MAM isolated from young individuals) [167]. In response to the oxidative stress, high p66Shc Ser36-phosphorylation status promotes both an additional intracellular ROS generation and reduction of the antioxidant defence system efficiency. The disturbance of the antioxidant enzymes level is connected with the recruitment of Akt to the p66Shc-FOXO3a complex, resulting in a direct inactivation of FOXO transcription factors by their phosphorylation [168, 169]. In this way, genes encoding antioxidant enzymes controlled by FOXO transcription factors are downregulated, with consequent increased ROS production and oxidative stress. Accordingly, decrease of phosphorylated p66Shc is accompanied by positive modulation of the antioxidant defence system [170].

Many alterations that extend lifespan affect not only stress response proteins, such as p66shc, but also nutrient sensors, such as insulin growth factor (IGF-1), target of rapamycin (TOR) protein kinase, AMP kinase (AMPK), sirtuins, and PGC-1*α* [171]. Caloric restriction (CR) is the best example of signals that modulate the activity of nutrients sensors, and it is the most robust intervention to extend lifespan and ameliorate various diseases in mammals, reducing oxidative stress and damage [172, 173]. Despite CR was historically considered a nongenetic process regulating lifespan, many recent lines of evidence have suggested its role in several signaling pathways, causing ROS reduction and mitochondrial biogenesis [174].

Nutrient sensor IGF-1 signaling pathway was the first to be associated to CR-mediated regulation of lifespan in animals. Indeed, heterozygous knockout mice for the IGF-1 receptor (Igfr +/−) live longer, show a delay in the onset of pathologies, and display greater resistance to oxidative stress [175, 176], suggesting that the effects of the IGF-1 pathway on longevity are closely related to mitochondrial protection from oxidative damage. Accordingly in *C. elegans*, mutations of downstream components of this pathway, such as age-1 (the homologue of PI3K) and akt-1 and akt-2 (the homologue of AKT1 and AKT2), result in extension of lifespan [153].

The most important targets regulated by IGF-1-signaling pathway are the transcription factors FoxO [177] and TOR protein kinase [178]. FoxO is required for the antiaging effects of the IGF-1 pathway [179, 180], once downregulated by CR, since it induces the expression of several antioxidative enzymes [181, 182], mediating the production of secondary signals that regulates lifespan.

TOR is highly conserved during evolution and functions as the major amino acid and nutrient sensor in the cell [183]. Much evidence points out the central role of this kinase in lifespan extension by CR. In yeast, *C. elegans,* and *Drosophila*, TOR is required for the effects of dietary restriction, and, importantly, its downregulation extends lifespan of all these models [184] and in mice too [185].

Recently, other signaling proteins have been shown to converge and regulate the FoxO transcription factors and thus oxidative stress and aging. Specifically, the proteins known as sirtuins, NAD-dependent histone deacetylases [186], are activated during CR, when the NAD/NADH ratio is elevated [187]. This group of proteins increases lifespan in yeast, *C. elegans*, and *Drosophila*, and it is thought to act similarly in mammals [188]. In this respect, aging is often associated with reduced sirtuins levels. Sirtuins are mainly antiaging genes via the promotion of mitochondrial function and autophagy and inhibition of apoptosis. They also have an inhibitory activity on ROS. Sirtuin function may be enhanced by restricting caloric intake or increasing physical activity, thereby, extending lifespan. Importantly, three of seven mammalian sirtuins (SIRT3, 4, and 5) are targeted to mitochondria, and SIRT1 is a regulator of mitochondrial biogenesis [189]. SIRT3 is required for the maintenance of mitochondrial integrity and metabolism during stress [190]. The protective effects of CR on oxidative stress and damage are diminished in mice lacking SIRT3. SIRT3 reduces cellular ROS levels dependent on SOD2. SIRT3 deacetylates two critical lysine residues on SOD2 (Lys^53/89^) and promotes its antioxidative activity. Importantly, the ability of SOD2 to reduce cellular ROS and promote oxidative stress resistance is greatly enhanced by SIRT3 [191]. In a very recent paper, Chen et al. have reported that SOD2 is also acetylated at Lys^68^ and that this acetylation decreases its activity. SIRT3 binds to, deacetylates, and activates SOD2. Increase of ROS levels stimulates SIRT3 transcription, leading to SOD2 deacetylation and activation [192].

Other studies confirmed that SIRT1 is part of the insulin/IGF-1 pathway, being able to deacetylate and, thus, activate FoxO transcription factors [193]. Moreover, SIRT1 can activate PGC-1*α* (peroxisome-proliferator-activated receptor *γ* coactivator-1*α*). Several data linked PGC-1*α* and aging. First, PGC-1*α* is progressively downregulated during aging, and this event is prevented by CR [194]. Furthermore, oxidative stress is known to induce the expression of this gene [195]. In general, PGC-1*α* improves mitochondrial dysfunction, the major phenotype of aging and age-related diseases, in a tissue-dependent fashion [196] and during CR promotes mitochondrial biogenesis [197], thereby lowering ROS production [198]. PGC-1*α* is also directly activated by phosphorylation by AMPK that is the master nutrient sensor in the cell [199]. The same kinase is able to phosphorylate FoxO factors too [200].

Recently another protein, named Bmi1, has demonstrated to increase the expression of a collection of gene products involved in mitochondrial function and ROS production in aging. Cells defective in Bmi1 have significant mitochondrial dysfunction with a sustained increase in ROS that is sufficient to cause a marked increase in the intracellular levels of reactive oxygen species and subsequent engagement of the DNA damage response pathway. Also mice Bmi1 −/− present numerous abnormalities including a severe defect in stem cell self-renewal, alterations in thymocyte maturation, and a shortened lifespan [201].

Aging is characterized also by the decline of the autophagic pathway [202]. As described in the previous section, autophagy is required in order to remove compromised mitochondria suffering from oxidative stress. Moreover, autophagy modulation in different model organisms has yielded very promising results suggesting that the maintenance of a proper autophagic activity contributes to extend longevity [203]. During aging, the number of mitochondria suffering from oxidative stress may increase, while their cleanup by the autophagic system may become limiting, leading to the accumulation of damaged mitochondria [204]. It is important to point out that a greater pool of functional mitochondria could ameliorate tissue damage by opposing cells against the gradual energetic decline occurring in cells as mitochondria become damaged during aging [171].

In this respect, the efficient and selective removal of damaged mitochondria by autophagy is a crucial element in the maintenance of cellular health since the accumulation of ROS from dysfunctional mitochondria and eventual cell death via apoptosis is avoided [204]. At the level of the organism, apoptosis will be the ultimate resort to remove seriously damaged cells. This will particularly affect the lifespan of nondividing cells, like neurons, thereby, affecting the lifespan of the whole organism. Indeed, the degenerative processes that arise in old organisms are partly caused by increased cell death of nondividing cells [205]. In any case, the accumulation of damaged mitochondria and their impaired removal is a hallmark of aging and will contribute to decreased cell viability. Interestingly, autophagy is regulated by the same signaling pathways that determine lifespan, acting as one of the downstream effectors of these signaling cascades. Autophagy is essential for the effects of the IGF-1/insulin pathway on longevity. The insulin/IGF-1 pathway regulates autophagy through TOR and FoxO, with opposite effects; TOR was shown to inhibit autophagy [206, 207], while FoxO induces autophagy [208, 209]. Moreover, SIRT1 seems to be, at least in part, responsible for the direct induction of autophagy [210], as it forms complexes with several autophagy-related genes (ATG) proteins, causing their deacetylation and activation [211]. Autophagy is suggested to be crucial also for the onset of age-related diseases since knockout animals for ATG5 and ATG7 in the nervous system show the accumulation of abnormal proteins and massive neuronal loss leading to neurodegeneration with decreased lifespan [212, 213]. Altogether, these data suggest the presence in the cell of a redundant control of autophagy that allows to finely tune this process under a wide array of physiological and pathological conditions.

## 7. Concluding Remarks

Mitochondria and ROS signalling tightly control cellular homeostasis by regulating fundamental cell-death and cell-survival processes like apoptosis and autophagy. Therefore, ROS production may be considered a “balance of power,” directing the cell towards life or death. It is clear that many proteins that mediate apoptosis and autophagy directly affect ROS signalling, through translocation to the mitochondrial compartment and/or modulation of pro/antioxidant proteins. Although correlation between mitochondrial ROS and aging has been the subject of debate for over forty years, recent discoveries about the role of autophagy in cleaning up damaged mitochondria, prolonging lifespan, underline the importance of studying ROS dynamics and show that they continue to be extremely fashionable research targets.
